# Identifying common treatments from Electronic Health Records with missing information. An application to breast cancer

**DOI:** 10.1371/journal.pone.0244004

**Published:** 2020-12-29

**Authors:** Onintze Zaballa, Aritz Pérez, Elisa Gómez Inhiesto, Teresa Acaiturri Ayesta, Jose A. Lozano

**Affiliations:** 1 BCAM - Basque Center for Applied Mathematics, Bilbao, Spain; 2 Hospital Universitario Cruces, Barakaldo, Spain; 3 Department of Computer Science and Artificial Intelligence, Intelligent Systems Group, University of the Basque Country UPV/EHU, San Sebastián, Spain; University of Oxford, UNITED KINGDOM

## Abstract

The aim of this paper is to analyze the sequence of actions in the health system associated with a particular disease. In order to do that, using Electronic Health Records, we define a general methodology that allows us to: (*i*) identify the actions in the health system associated with a disease; (*ii*) identify those patients with a complete treatment for the disease; (*iii*) and discover common treatment pathways followed by the patients with a specific diagnosis. The methodology takes into account the characteristics of the EHRs, such as record heterogeneity and missing information. As an example, we use the proposed methodology to analyze breast cancer disease. For this diagnosis, 5 groups of treatments, which fit in with medical practice guidelines and expert knowledge, were obtained.

## Introduction

**Fig 1:**
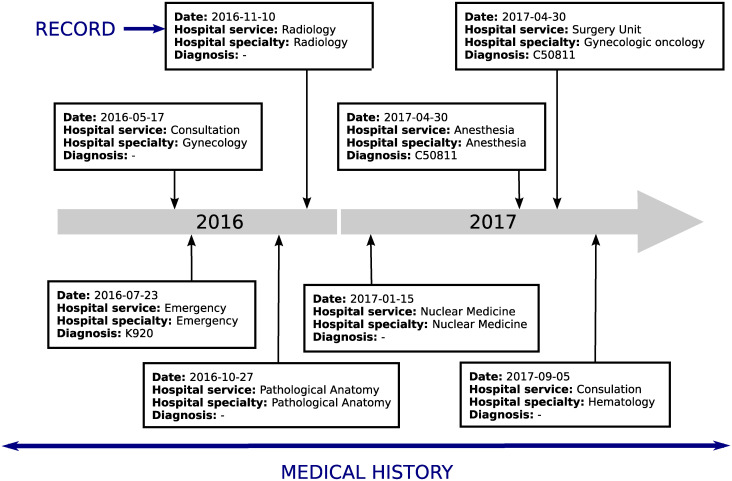
A simplified EHR structure.

Electronic Health Records (EHRs) are defined as an electronic collection of medical information about the health histories of patients, such as diagnosis, drugs, tests, allergies, and so on [1]. We can observe a simplified example of this type of database in Fig 1. Each row (or record) is made by a medical event that gathers information about the activity performed: patient ID, date, visited hospital service, visited medical specialty, diagnosis and procedure. A suitable manner of representing a medical history of a patient is as a sequence of discrete actions in the health system [2], where the order of events can contain relevant information about the treatment.

The aim of this paper is, using EHRs, to analyze the sequence of medical actions in the health system associated with a particular disease. Specifically, for each patient diagnosed with the disease, we would like to extract the sequence of medical actions associated with the complete treatment of it.

In order to do that, we design a specific methodology that takes into account the challenging characteristics of the EHRs [3]:

- Heterogeneity: The EHR data contains a large amount of distinct medical events (e.g., diagnosis, medication, lab).
- Incomplete information: This is a common characteristic in EHRs, where missing values frequently outnumber observed values. In particular, we use a real-world administrative database characterized by the huge number of missing values in the diagnosis variable (75%).

**Fig 2:**
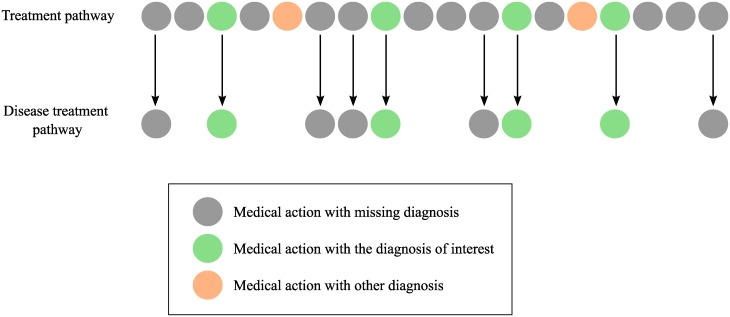
Ambiguity of medical actions associated with a diagnosis due to comorbidity and missing values.

This lack of diagnosis values in many medical actions, together with the comorbidity of many patients, creates uncertainty about whether a medical action is associated with a particular disease or not (see Fig 2). In other words, patients are likely to have co-existing diseases, consequently, some actions are unknown which disease treatment pathway they belong to because their diagnosis value is missing in the EHRs.

In light of the above, we propose a general methodology that allows us to: (*i*) identify the actions in the health system associated with a disease; (*ii*) identify those patients with complete treatment of the disease; (*iii*) and discover common treatment pathways followed by the patients with a specific diagnosis. As an example in a real scenario, we use the methodology to analyze breast cancer disease. The outcomes are compared with clinical practice guidelines to show if the theory and reality match, or, on the contrary, there exist any deviation in practice.

### Motivation

Previous studies have focused on the identification of clinical pathways and treatment patterns from EHRs using process mining [4–7]. The basic idea of process mining is to extract knowledge from event logs, and in the healthcare domain, medical activities from EHRs are used as process logs [8]. However, most of the health data have diverse behavior and are not well structured. This assumption leads to spaghetti-like workflow models that are very difficult to interpet [7].

Machine learning techniques provide a potential solution to these spaghetti models by grouping patients in relatively homogeneous subgroups. For example, in [9], prior to treatment pathway extraction, the authors used hierarchical clustering with longest common subsequence distance to measure similarity between sequences. In [10] patients were segmented by their outcomes, followed by further clustering using DBScan with Leveinshtein distance, and frequent pattern mining using the SPAM algorithm. In [11] the authors applied fuzzy c-means in order to group the patients according to some selected statistical characteristics. Next, the treatment pathway data were used to generate frequent episodes that characterize each group. Finally, [12] employed K-means together with Leveinshtein distance to obtain subgroups of patients. Subsequently, the typical clinical pathways were represented by directed graphs with the edges weighted according to the flow of each cluster.

These models do not deal with common challenges of clinical data such as inaccuracy, incompleteness, comorbidities, active treatments that provide just a partial view of the entire medical history, and so on. Our model is primarily focused on these matters in order to obtain complete treatment pathways for an accurate analysis of the disease. The second reason for developing a new methodology is associated with the identification and representation of the different treatment pathways. There are some approaches that, after grouping the points using a clustering technique, depict the subgroups by the most frequent medical activities instead of the whole set of activities implied [9, 12]. That is, they are represented by a partial set of medical activities rather than by a real and complete treatment pathway. Some other techniques segment the patients by common medical characteristics such as diagnosis or outcomes, and they subsequently construct the typical treatment pathways within each group [10, 11]. However, our methodology groups the treatment pathways in subgroups based on their actions and the order in which they occurred. In addition, it enables the representatives of the clusters to be depicted by a real treatment pathway of the disease of interest.

## Materials and methodology

### Materials

The study is performed on a dataset from the public health care system of the Basque Country (Spain) called Osakidetza. This is a database recording the medical histories of 579.798 patients concerning different levels of healthcare (1 hospital, 11 outpatient clinics and emergency care) in 2016 and 2017. Remember that the aim of the study is to analyze the sequence of actions in the health system, therefore, the treatments will be represented as pathways followed in the hospital. For that reason, henceforth, we will only consider hospital services, medical specialties and diagnosis information. Then, we denote an **action** as a tuple *a* = (*s*, *m*, *d*) where *s* refers to the hospital service visited, *m* refers to the medical specialty and *d* refers to the diagnosis. Hence, a **treatment pathway** of a patient is an ordered sequence of actions *A* = *a*_1_ *a*_2_…*a*_*m*_.

These treatment pathways are not associated with a unique disease due to the existence of comorbidity in the patients (see Fig 2). An example is a patient with a diagnosis of diabetes mellitus and lung cancer. When the diagnosis value is missing, we can not assure if the medical action is related to the cancer or to the diabetes condition. Therefore, if we are able to assign each action to a disease, the treatment pathway of a patient can be seen as a set of subsequences of actions associated with different diseases. We refer to the subsequence of actions associated with a diagnosis as **disease treatment pathway**.

### Methodology

**Fig 3:**
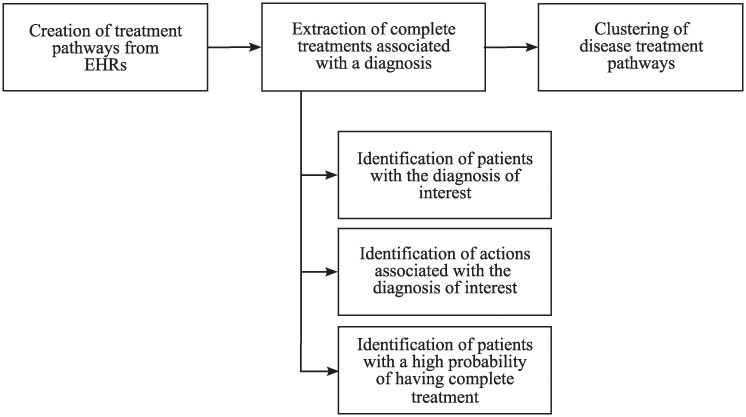
Methodology of the study.

In this section, we present the methodology (Fig 3) to identify the different treatments of a disease followed by the patients in the health system. For this purpose, we developed a methodology to extract complete treatments associated with a diagnosis from the entire medical history of the patients. Afterwards, we applied a clustering method with the aim of identifying the different groups of disease treatment pathways. Note that clustering is an unsupervised technique and it is performed without prior knowledge about the disease. Therefore, although the validation of the clusters could be carried out in terms of compactness or coherence, we thought that the most appropriate evaluation of our approach was by checking the results with medical guidelines and physicians. We proceed in this way to validate the applicability of the whole methodology.

#### Creation of treatment pathways from EHRs

We need to convert the original EHRs (Fig 1) into treatment pathways. As aforementioned, this structure is based on creating a sequence of actions from the records of the database in such a way that each patient has an associated treatment pathway. Therefore, we work on discrete sequences, variable in length because of the great deal of heterogeneity in the medical histories of the patients. For instance, there may exist a patient with only two visits to the hospital just for check ups, whereas another patient suffering from a chronic disease regularly visits the hospital due to therapy, analytics, tests and so on.

#### Extraction of complete treatments associated with a diagnosis

The next goal is the extraction of complete treatment pathways associated with a diagnosis from the EHRs, which is merely the extraction of the subsequence of actions associated with the diagnosis of interest, that is, the disease treatment pathway. Firstly, we identify the patients with the diagnosis of interest recorded. Then, to cope with the lack of diagnosis information and comorbidity in many patients, we determine which actions of the patients are associated with the pathology in order to avoid typical actions of another diagnosis in the disease treatment pathway. Once these pathways are created, we have to select the patients with a high probability of having recorded the complete treatment of the disease, and, eventually, we will be able to obtain the disease treatment pathways of interest from EHRs.

*Identification of actions associated with a diagnosis*. Due to the missing values in the diagnosis together with the comorbidity in patients, we are not able to directly extract the disease treatment pathway. Hence, we propose a relevance measure to identify which are the typical actions related to the diagnosis of interest within a treatment pathway (see Fig 2). In order to do that, we first divide the database into two groups of patients: patients with at least one action with the diagnosis of interest, and patients without it. Then, we check the medical specialty in which the action occurs. We calculate the mean frequency of the medical specialty in both groups, and the relevance is defined as the ratio of the mean frequency between the groups. Now, the higher the relevance is, the more important the action is for the disease. Therefore, we establish a threshold *τ* in such a way that if the relevance is higher than *τ*, we include the action in the disease treatment pathway. That is, an action is typical of the disease if

$$\begin{array}{c} \frac{f_{m_{D}}}{f_{m_{R}}}\geq \tau , \end{array}$$ (1)

where $f_{m_{D}}$ and $f_{m_{R}}$ are the mean frequency of the times attended to a medical specialty in the patients with the targeted diagnosis and the rest of the patients, respectively.

*Identification of patients with complete treatments*. As previously mentioned, the first step was to identify patients with the diagnosis of interest recorded in the treatment pathways. However, it is not sufficient when it comes to obtaining complete treatments for various reasons: pathways might contain actions of similar diseases (e.g., different types of cancer) that make it difficult to know on which diagnosis the treatment is focused; there might exist treatments which started previously to or finished later than the recording period; or even uncompleted treatments with lost follow-up. Hence, we propose some selection criteria to deal with these issues:

- Ensuring that the treatment pathway performed is directly focused on the aimed diagnosis: a requirement to ignore patients with similar coexisting diseases (and therefore, treatments) recorded in their medical histories.
- Avoiding treatments started before the recording period of the database or treatments which did not finish before the closing date: medical procedures that are essential to diagnose a disease must be required in every disease treatment pathway. Likewise, having no diagnosis-related actions in the first and last months of the recording period is an important requisite to obtain complete treatments.
- Avoiding treatments with incomplete follow-up: a minimum follow-up time and a minimum amount of actions recorded are essential.

These selection criteria must be adjusted specifically to each disease, taking into account that the initial or final actions, as well as the typical timestamps between initial and final actions, are different depending on the diagnosis that was sought after. Once defined, the criteria are applied one by one to the data in order to filter out the patients that do not satisfy the requirements. The main reason for this data reduction is to select the patients that, with a high probability, have the complete treatment of the disease recorded in the data.

Summarizing, we achieve the following objectives with the proposed methodology: (*i*) identifying the actions associated with a diagnosis, and therefore, the disease treatment pathway of the diagnosis of interest by deleting the actions related to other coexisting diseases; (*ii*) selecting the patients with a high probability of having the full treatment of the diagnosis recorded in the data by applying the selection criteria. In other words, we are able to extract the complete treatments made up by actions associated with the diagnosis of interest. After that, the candidates are ready to be grouped in order to discover the common treatment patterns of the disease.

#### Clustering: K-medoids with edit distance

Once the pathways of treatments associated with the diagnosis of interest are selected, that is the sequences that represent complete treatments, we proceed to identify the most significant groups of treatments. Here, the main idea is to group together treatments in such a way that those within a group are similar to each other but are dissimilar to treatments assigned to other groups. Therefore, the clustering method [13] seems to be a logical and promising approach.

However, we should previously select a suitable distance measure that enables the comparison of discrete action sequences with variable lengths. For this purpose, the most commonly used sequence distance is the Levenshtein distance [14], which enables us to calculate the similarity (or dissimilarity) between two sequences, and it is defined as follows.

Given two strings *A*_1_ and *A*_2_ over a finite alphabet, the edit distance between *A*_1_ and *A*_2_ can be defined as the minimum weight of transforming *A*_1_ into *A*_2_ through a sequence of weighted edit operations. These operations are usually defined in terms of insertion, deletion, and substitution of one symbol for another, possibly with different costs for each of these operations. In this work, the cost of insertion and deletion is 1, whereas the cost of substitution is 2. Nevertheless, the edit distance is not sufficient for many applications comparing strings with different lengths. Hence, normalization should be applied to appropriately rate the weight of the edit errors concerning the sizes of the objects that are compared [14, 15].

Finally, in order to generate groups of action sequences taking into account their distances, we make use of K-medoids clustering method [16] which is a variance of K-means algorithm but more appropriate for making clusters of sequences of actions for serveral reasons: i) it can be computed using distances between every pair of sequences of actions; ii) it does not require to compute the centroid of a given set of sequences which is computationally intractable and can generate senseless sequences; iii) each cluster of sequences is characterized by a real sequence of actions, called the medoid; and iv) it is more robust to noise and outliers.

The medoid sequence of a cluster is defined as the sequence of the cluster that has the lowest average distance to the rest of the sequences belonging to the cluster. In particular, Partitioning Around Medoids [17] is a representative K-medoids clustering algorithm. The basic idea is as follows: it searches for *k* representative objects in a data set (*k* medoids) and then assigns each object to the closest medoid in order to create clusters. Its aim is to minimize the sum of dissimilarities between the objects in a cluster and the medoid of the same cluster.

- *Step 1*. Initial step: arbitrarily choose *k* of the *n* data points as the medoids to form initial clusters.
- *Step 2*. Assignment step: associate each data point to the closest medoid.
- *Step 3*. Update step: for each medoid *m* and each data point *x* associated to *m*, swap *m* and *x* and compute the average dissimilarity of *x* to all the data points associated with *m*. Select the medoid *x* with the lowest average dissimilarity.
  Repeat alternating steps 2 and 3 until there is no change in the assignments.

Thus, with K-medoids method we avoid creating artificial sequences of actions for characterizing each group because the representative sequences are real sequences belonging to the database. Besides, obtaining these action sequences that minimize the mean distance relative to the rest of the sequences of the group is an NP-hard problem.

## Results

For the validation of the proposed methodology to generate groups of treatments for a given diagnosis, we will use breast cancer patients as a case study using the dataset provided by Osakidetza.

### Extraction of complete treatments associated with breast cancer

First of all, the target population comprised 1456 patients with breast cancer diagnosis out of 579.798 patients between January 1, 2016, and December 31, 2017. This selection of patients from the database is made according to the *International Statistical Classification of Diseases and Related Health Problems (10th revision)* [18], where every code starting by *C50* corresponds to breast cancer diagnosis.

#### Identification of actions associated with breast cancer

Remember that the association of actions with a diagnosis is made through the relevance of the medical specialties (Eq 1). Table 1 shows those medical specialties whose relevance is higher than *τ* = 3. Only actions carried out in these 18 medical specialties are included when creating the final treatment pathways of patients with breast cancer, but once they are extracted, 21 patients out of 1456 had no action which occurred in these medical specialties, therefore, they are excluded from the study.

**Table 1:** Relevance of the medical specialties associated with breast cancer diagnosis.

Medical Specialties	Relevance	Medical Specialties	Relevance
Gynecologic Oncology	85,9	Gynecology	9,3
Radiotherapy	78,3	Genetic Laboratory	8,8
Plastic Surgery	66,0	Surgery Unit	5,8
Medical Oncology	36,4	Anesthesia	5,6
Day Hospital	16,5	Home Hospitalization	4,8
Nuclear Medicine	11,7	Pathological Anatomy	4,2
Day Surgical Hospital	10,5	Hospitalization	3,5
Genetics	10,2	Others	3,3
Major Burns Unit	9,5	Radiology	3,0

Table 1 shows the relevance of the medical speciatlies given at least 3 times more frequently in breast cancer patients. These are the ones to be considered to create the breast cancer treatment pathways of patients.

#### Identification of patients with complete treatments

**Fig 4:**
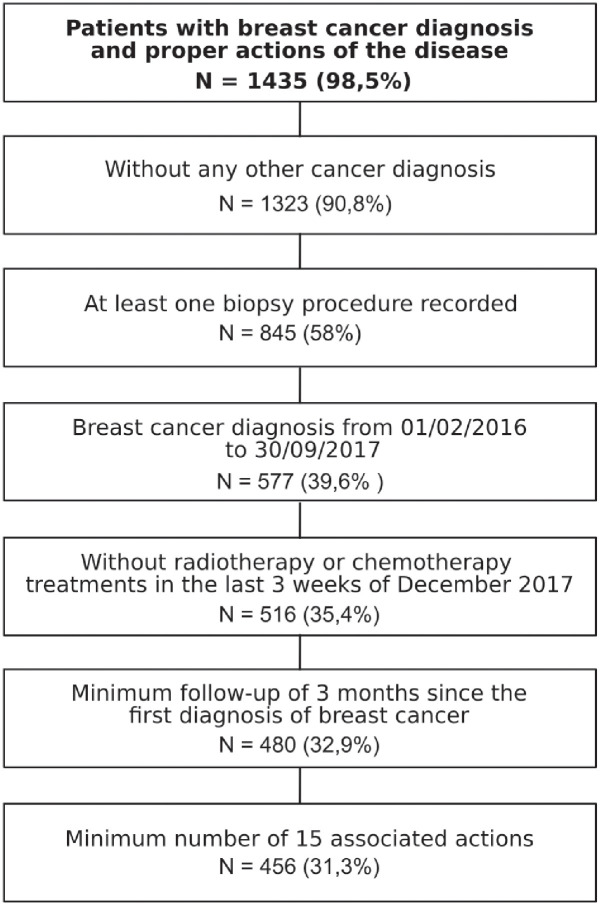
Proposal for the selection criteria of breast cancer diagnosis.

Once the association between actions and breast cancer diagnosis is known, we can extract for each patient the subsequence of actions that describe the treatment of breast cancer. However, these sequences may be incomplete. Hence, we will select the sequences of actions that have high probability of describing complete treatment pathways of breast cancer. In order to do that, we propose some selection criteria, listed in Fig 4 and explained as follows.

First of all, the patients with any other type of cancer diagnosis apart from breast cancer are filtered out, otherwise, we could not distinguish which cancer diagnosis the treatment is focused on. Moreover, to ensure that the pathology has been diagnosed in the recording period of our database, at least one record of a breast biopsy procedure is required. It is the only definitive diagnostic procedure to determine if the suspicious area is cancerous [19], and therefore, should be performed for every breast cancer diagnosed patient.

Regarding the recording time of treatments, we consider that a treatment is completely recorded in the database if there is no diagnosis in the first and last months. Therefore, the breast cancer diagnosis must be between the 1st February 2016 and the 30th September 2017. If any patient with a breast cancer diagnosis record out of this period was included, we assume that it is the continuation of the treatment previously started or the continuation after 2017.

For the same reason, we need to avoid radiotherapy or chemotherapy actions in the last period of the database. Radiotherapy is delivered daily or every 2 days, and chemotherapy every 1-3 weeks [19]. Therefore, if there exists any radiotherapy or chemotherapy action in the last 3 weeks of 2017, it means that it is an unfinished treatment.

Likewise, the period of medical assistance recorded must be at least 3 months once the patient has been diagnosed with breast cancer. Additionally, the minimum number of associated actions in their treatment pathways must be at least 15 in order to avoid incomplete sequences of actions, this could mean that patients abandoned the treatment or their follow-up was lost for some reason.

**Fig 5:**
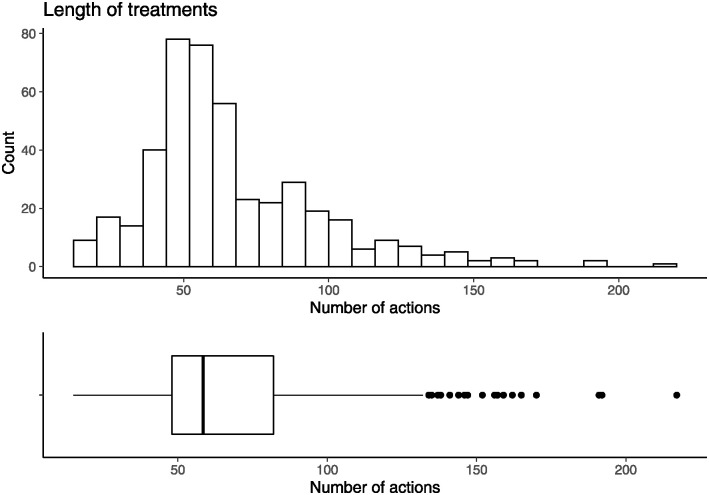
Distribution of the length of the breast cancer treatment pathways.

After applying these selection criteria, there are in total 440 out of 1456 patients (31.3%) with a high probability to present a complete treatment of breast cancer in our EHRs. These breast cancer treatment pathways are made up of the actions occurred in the aforementioned medical specialties and they are the sequences of actions to be grouped. The treatment pathways are of variable lengths, in fact, the minimum treatment pathway is made of 15 actions and the maximum one of 217 actions. The distribution of these durations of treatments is shown in Fig 5.

### Representative sequences and clinical practice guidelines

**Fig 6:**
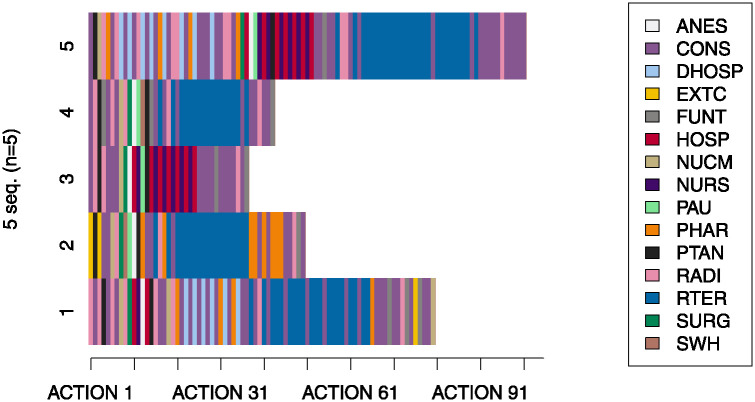
Clustering results. Representative medoids considering 5 groups. ANES: Anesthesia; CONS: Consultation; DHOSP: Day Hospital; EXTC: External Consultation; FUNT: Functional Testing; HOSP: Hospitalization; NUCM: Nuclear Medicine; NURS: Nursing; PAU: Post Anesthesia Care Unit; PHAR: Pharmacy; PTAN: Pathological Anatomy; RADI: Radiology; RTER: Radiotherapy; SURG: Surgery; SWH: Surgery Without Hospitalization.

K-medoids algorithm was applied to the selected disease treatment pathways in order to identify the treatment patterns of breast cancer patients, and the selection of K was checked from 2 to 10. From 5 clusters on, the treatment patterns were repeated, and therefore, we decided to create a total of 5 groups, which are shown in Fig 6. On the one hand, the 5 horizontal lines are the representative disease treatment pathways (medoids), and, on the other hand, the vertical lines correspond to the hospital services visited by the representative patients in each action.

To validate the results, the representative pathways were compared with clinical practice guidelines, specifically, with the *European Society for Medical Oncology* breast cancer guideline [19, 20]. These guidelines provide updated state-of-the-art recommendations on management of breast cancer (diagnosis, treatment and follow-up). Besides, the outcomes were also contrasted and approved by physicians.

The 5 sequences obtained fundamentally represent different treatment pathways to deal with breast cancer. We can see in Fig 6 that all of them start with Consultation, Pathological Anatomy, Nuclear Medicine and Radiology visits. In these hospital services, the breast examinations and tests are carried out: in Radiology tests such as sonography, mammogram or even some radiography; in the case of Pathological Anatomy and Nuclear Medicine, the biopsy test and cancer diagnosis. According to the clinical practice guideline, a biopsy must be done before any type of treatment is initiated and the five groups accomplish it in Pathological Anatomy actions.

**Fig 7:**
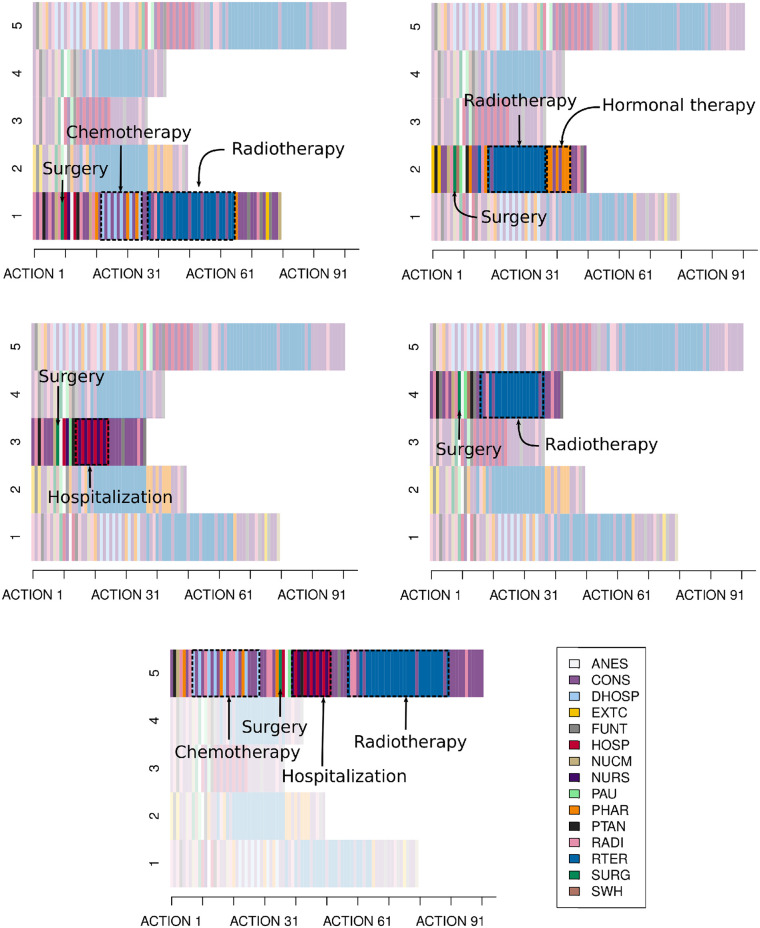
Clustering results. Treatment patterns of the groups.

The main therapies of each group are as follows (Fig 7):

- **Group 1** (66 patients, 15%).
  - Treatment pattern: Surgery + Chemotherapy + Radiotherapy.
  - Representative disease treatment pathway: The representative disease treatment pathway is administered by chemotherapy for 15 weeks (the recommended duration is 12-24 weeks) after breast-conserving surgery, and then, a month of radiotherapy is delivered. According to the guideline suggestions, if both therapies are used, chemotherapy should usually precede radiotherapy, as done here.
- **Group 2** (89 patients, 20.3%).
  - Treatment pattern Surgery + Radiotherapy + Hormonal Therapy.
  - Representative disease treatment pathway: This representative patient combines radiotherapy and hormonal therapy. The medical guideline mentions that hormonal therapy can be delivered safely with radiotherapy and normally lasts 5-10 years. This follow-up cannot be corroborated since the database gathers information over a period of up to 2 years.
  Hitherto, it is worth mentioning that there exist two types of surgery when it comes to breast cancer: breast-conserving surgery, in which the surgical team removes the tumor but tries to keep as much of the breast as possible (it is the preferred local treatment option for the majority of early breast cancer patients, in fact, this procedure is performed in most of the groups); or mastectomy, in which the whole breast is removed. In this latter case it is possible to have no therapy after surgery, and in general terms, these are commonly early invasive breast cancer patients [19].
- **Group 3** (108 patients, 24.6%).
  - Treatment pattern: Surgery + Hospitalization.
  - Representative disease treatment pathway: we suspect that it corresponds to the group of patients undergoing mastectomy, since they have no therapy after the surgical procedure, just a sequence of hospitalization actions combined with nursing actions. These hospitalizations after undergoing surgery are probably due to complications, that is, deviations from guidelines since nothing is explicitly mentioned there about hospital stays.
  - It is one of the groups with the highest number of patients, however, we suspect that some of these patients come from other hospitals just to undergo surgical treatment. We reached this conclusion because, according to practitioners and clinicians, it is not quite common to have such a big number of patients without therapy after surgery.
- **Group 4** (137 patients, 31.2%).
  - Treatment pattern: Surgery + Radiotherapy.
  - Representative disease treatment pathway: The representative patient undergoes breast-conserving surgery, and then receives postoperative radiotherapy, which is highly recommended in practice guidelines. This is the most simple and common delivered treatment.
  Until now, all the representative treatments start therapy after undergoing surgery, which is called Adjuvant Systemic Treatment. However, the remaining group is the only one that also receives therapy before undergoing surgery. This type of treatment is called Neoadjuvant Systemic Treatment and should be used to reduce the extent of surgery in locally advanced and large operable cancers.
- **Group 5** (40 patients, 9.1%).
  - Treatment pattern: Chemotherapy + Surgery + Hospitalization + Radiotherapy.
  - Representative disease treatment pathway: According to the guidelines, when Neoadjuvant Systemic Treatment is used, all chemotherapy should be delivered preoperatively as done here. In particular, 8 rounds of chemotherapy were delivered in 16 weeks, which comes with the recommendation of 12-24 weeks. Furthermore, they mention that magnetic resonance imaging of the breast, which is a test used to detect breast cancer and other abnormalities, is the most accurate modality for assessing the extent of residual disease following Neoadjuvant Systemic Treatment. It should also be carried out before initializing the treatment for proper comparative evaluation. In this patient they mention it was carried out in the Radiology unit after the 5 first sessions of chemotherapy and once the therapy was finished. After breast-conserving surgery, postoperative radiotherapy was delivered, strongly recommended by the clinical guideline.
  - We can observe also in this group some hospitalization actions that do not come with medical practice guidelines.

The follow-up of the patients is not clearly defined since our database only covers 2 years. However, in these 2 years, based on the clinical guideline recommendations, regular visits should be made every 3-4 months. These regular visits correspond to Consultations in the final part of the representative disease treatment pathways. Furthermore, annual bilateral (after breast-conserving treatment) and/or contralateral mammography (after mastectomy) is also recommended. Bilateral mammography in Radiology was performed in the 5 groups. In some cases, they also have Functional Testing actions (groups 1, 2, 3 and 4) or Nuclear Medicine actions (group 1), which are also likely to be related to the follow-up.

## Discussion

The methodology was designed to tackle the missing information and heterogeneity of EHRs. In addition to that, we also faced the difficulty of having comorbidity together with missing diagnosis. Its applicability and effectiveness were tested with breast cancer patients, however, it can be directly applied to identify the different treatment patterns of any other pathology, even for short-duration diseases. Subsequently, a comparison of the outcomes with clinical practice guidelines can be carried out in order to conclude whether they are actually followed in practice or not. It is also worth mentioning that the obtained treatment patterns might be useful for identifying deviations in the treatments from practice guidelines.

There exist also some limitations in the application of the proposed methodology. On the one hand, common diagnoses are likely to end up in failure when identifying associated actions, for example, a diagnosis of acute sinusitis. Patients with this type of usual pathologies may visit regular medical specialists (e.g., primary care or consultations), and therefore are unlikely to present high relevance values. That is, they will have no distinctive action in order to extract the associated disease treatment pathways (see Eq 1). In these particular cases, we should ask experts about the most common medical specialties for treating the disease, and afterwards, the clustering would work properly.

On the other hand, another weakness in the extraction of complete treatments from EHRs appears when the aimed diseases are of long-duration treatment (longer than the recording time of the database). These pathologies will have no complete treatments in the dataset as required in the proposed methodology. In fact, in the particular case of breast cancer, some treatments usually finish with hormonal therapy for 5-10 years, however, the recording time of the dataset is of 2 years. For this reason, we propose a future line of work to resolve this issue, similar to [21]: the design of a method for creating complete treatments of pseudopatients by merging partial treatments. In other words, it consists of aligning the final part of some patients’ disease treatment pathways that coincide, to some extent, with the initial part of others.

With regard to the selected clustering method and in comparison with other clustering algorithms, PAM has the drawback of working inefficiently for medium and large data sets. In fact, the complexity of the algorithm is *O*(*n*^2^) just in one iteration. Thus, it is obvious that PAM becomes too costly for large values of *n*. However, there exist algorithms that improve the performance of PAM, such as CLARA and CLARANS [22]. CLARA repeatedly applies PAM on a subsample of the data set and the remaining objects are assigned to their closest medoid. CLARANS works on the entire data set, but only explores a subset of the possible swaps of medoids and non-medoids using sampling.

Furthermore, the time variable is not being exploited in the proposed methodology. Involving this variable in the methodology might improve the results in several ways [23, 24]: firstly, in the identification of actions associated with the diagnosis; and secondly, the clustering outcomes would be purer and more homogeneous. We could take advantange of it by including the timestamp in the definition of an action as Δ*t* = *t*_*i*_ − *t*_*i*−1_, and then, any action with a Δ*t* value higher than a threshold *τ* will not be included in the disease treatment pathways. For instance, in the case of breast cancer, it makes no sense to have a surgical action without any prior breast cancer-related action (e.g., a biopsy procedure) within a period of 2 months. Likewise, the cluster outcomes might be improved if the time were considered when defining the proper distance for comparing sequences: the larger the Δ*t* value, the larger the penalization between actions, even if the hospital services match.

Finally, it would be interesting to include the relevance in the distance between sequences of actions. We computed the relevance as the ratio of mean frequencies of medical specialties visited in the group of patients with the diagnosis and in the rest of the patients. The ratio, which basically signifies how more frequently each medical specialty is given in the patients with the diagnosis, could be interpreted in terms of importance as something particular of the disease. Then, we could include the obtained relevance values in the edit distance as weights, similar to the Edit Distance with Real penalties [25]. Thus, actions considered proper of the disease would have a higher weight, and consequently, more importance when perfoming the clustering of sequences.

## Conclusion

The EHRs collected from the hospital gathers medical information about 579.798 patients, and there therefore exists a great deal of knowledge to be extracted. This information is about the patient ID, date of visit, diagnosis, procedure, hospital service visited and medical specialty visited.

Since the objective of the study is to obtain treatment patterns for a given pathology, the first idea is to convert the EHRs into action sequences in such a way that the sequence is able to describe the treatment as a pathway followed in the hospital. Hence, an action is defined as a tuple of diagnosis, hospital services and medical specialty, and consequently, the sequence of actions is the treatment pathway followed by each patient.

However, these sequences are characterized by having missing information: 75% of the diagnosis variable are missing values and there exists comorbidity. Consequently, it is difficult to extract the sequence of actions associated with the disease of interest. Even so, once the subsequence is extracted, we do not know whether it is a complete or partial treatment.

Therefore, we designed a methodology which is able to cope with all these difficulties and able to obtain the representative treatment patterns from data. Firstly, we need to select only the actions associated with the diagnosis of interest. For this purpose, we defined a relevance measure for an action given in a pathology. Here, we divided the patients into two groups: patients with at least one record with the diagnosis and patients without the diagnosis. Then, we calculated the mean frequency of each medical specialty for both groups. The relevance of an action for a given diagnosis is defined as the ratio between the mean frequency of an action of both groups. Now, a relevance value higher than *τ* implies that the action is proper of the disease. These actions are selected for creating the disease treatment pathways. Thus, we manage to ignore the actions not related to the diagnosis of interest. Nevertheless, this is not sufficient when it comes to obtaining complete treatments because these disease treatment pathways may be incomplete. Therefore, some selection criteria are defined to extract patients with high probability of having the complete treatment associated with the diagnosis of interest recorded in the data. Finally, to fulfill the objective of identifying the main representative treatment patterns, the patients are grouped by means of the K-medoids algorithm. We chose K-medoids mainly because it groups similar treatments in the same cluster, and because these groups are represented by real treatments. For this purpose, we propose the use of the edit distance, which is able to compare two discrete sequences of different length.

The easy applicability of the proposed methodology is worth highlighting as well as its adaptation to different pathologies. In fact, its performance was demonstrated in a real scenario applying the proposed methodology to breast cancer patients. Subsequently, the results were validated with physicians from Osakidetza.

The adherence to the *European Society for Medical Oncology* [19, 20] guidelines was checked with the outcomes obtained, a total of 5 representative sequences. With regard to therapies, the treatment patterns were the following ones:

- Surgery + Chemotherapy + Radiotherapy
- Surgery + Radiotherapy + Hormonal Therapy
- Surgery + Hospitalization
- Surgery + Radiotherapy
- Chemotherapy + Surgery + Hospitalization + Radiotherapy

In conclusion, the proposed methodology enables us to easily identify the treatment patterns of a pathology from EHRs, despite their missing information concerning the diagnosis. This is a common characteristic of clinical databases, which did not hinder reaching our ultimate objective, as was shown in the breast cancer analysis.
